# Ataxin-3 Plays a Role in Mouse Myogenic Differentiation through Regulation of Integrin Subunit Levels

**DOI:** 10.1371/journal.pone.0011728

**Published:** 2010-07-23

**Authors:** Maria do Carmo Costa, Fernanda Bajanca, Ana-João Rodrigues, Ricardo J. Tomé, Garry Corthals, Sandra Macedo-Ribeiro, Henry L. Paulson, Elsa Logarinho, Patrícia Maciel

**Affiliations:** 1 Life and Health Sciences Research Institute (ICVS), School of Health Sciences, University of Minho, Braga, Portugal; 2 Department of Neurology, University of Michigan, Ann Arbor, Michigan, United States of America; 3 Instituto de Biologia Molecular e Celular (IBMC), Universidade do Porto, Porto, Portugal; 4 Turku Center for Biotechnology, Turku, Finland; National University of Singapore, Singapore

## Abstract

**Background:**

During myogenesis several transcription factors and regulators of protein synthesis and assembly are rapidly degraded by the ubiquitin-proteasome system (UPS). Given the potential role of the deubiquitinating enzyme (DUB) ataxin-3 in the UPS, and the high expression of the murine ataxin-3 homolog in muscle during embryogenesis, we sought to define its role in muscle differentiation.

**Methodology/Principal Findings:**

Using immunofluorescence analysis, we found murine ataxin-3 (mATX3) to be highly expressed in the differentiated myotome of E9.5 mouse embryos. C2C12 myoblasts depleted of mATX3 by RNA interference exhibited a round morphology, cell misalignment, and a delay in differentiation following myogenesis induction. Interestingly, these cells showed a down-regulation of α5 and α7 integrin subunit levels both by immunoblotting and immunofluorescence. Mouse ATX3 was found to interact with α5 integrin subunit and to stabilize this protein by repressing its degradation through the UPS. Proteomic analysis of mATX3-depleted C2C12 cells revealed alteration of the levels of several proteins related to integrin signaling.

**Conclusions:**

Ataxin-3 is important for myogenesis through regulation of integrin subunit levels.

## Introduction

Myogenesis is the process of differentiation of muscle precursor cells (MPCs) into multinucleated myotubes that mature into myogenic fibers [1], [2]. This is strictly regulated by two families of transcription factors: the myogenic regulatory factors (MRFs), and the myocyte enhancer-binding factor 2 (MEF2). The MRFs, consisting of Myf5, MyoD, MRF4 and myogenin, usually forming heterodimers with ubiquitous E-proteins (E12 or E47) [3], act in combination with MEF2 transcription factor family to activate many muscle-specific genes [4].

For successful myogenesis many cellular processes must occur, directed by transcriptional regulation by MRFs and MEF2, intracellular signaling, cell migration, cell-cell interaction, and cell connections with the extracellular matrix (ECM). Integrins are important players in these processes. These transmembrane cell surface ECM receptors form heterodimers composed of an α and β subunits [5]. Clustering of integrins upon matrix binding provides a bidirectional crosstalk between the inside and outside signaling. As a consequence of cell-matrix adhesions, several proteins are recruited to the adhesion complexes, activating signaling cascades that control cytoskeletal organization, gene regulation, and diverse cellular processes and functions [6]. In vitro and in vivo studies in avian and rodent species showed that α4β1, αVβ1, α5β1, α6β1, and α7β1 integrins are the major players in skeletal muscle development [7].

It is crucial that the synthesis, degradation, and assembly of many proteins be closely regulated at each phase of muscle development. Many transcription factors and other proteins participating in the aforementioned processes are rapidly degraded by the UPS, including MyoD [8], Myf5 [9], myogenin [10], Pax3 [11], Id proteins [12], β-catenin [13], [14], and α5 integrin subunit [15].

The reversal of ubiquitination, catalyzed by deubiquitinating (DUB) enzymes, is equally important in several cellular processes. So far, only two DUBs, UBP45 and UBP69, have been implicated in myogenesis but their specific role is still unknown [16]. Thus, it remains to be determined how the removal of ubiquitin from certain proteins by DUBs contributes to muscle development.

Ataxin-3 (ATX3) is the best characterized member among a small new subclass of DUBs [17]. DUB activity in ATX3 is conferred by the N-terminal josephin domain, carrying the essential Cysteine 14 (C14) residue responsible for its protease activity [18], [19], [20]. Several lines of evidence suggest that ATX3 may function in the UPS: (1) it binds and hydrolyses polyubiquitin chains *in vitro* [21], [22], [23], [24]; (2) it interacts with ubiquitin (Ub) and NEDD8, a ubiquitin-like (UBL) protein that regulates the activity of E3 ligases [25]; and (3) it associates with the proteasome through interactions with p45 (an ATPase subunit of the 19S proteasome subunit) [26], VCP/p97 (an AAA ATPase important for the shuttling of many polyubiquitinated proteins to the proteasome) [27], [28], and Ubxn-5 [29].

ATX3, an ubiquitously expressed protein that contains a polyglutamine (polyQ) tract near its C-terminus that is neighbored by two or three ubiquitin interacting motifs (UIMs) [24], depending on the protein isoform. In humans, this polyQ segment usually consists of 12–44 glutamines, but it is expanded to ∼60–87 glutamines in Machado-Joseph disease (MJD), also known as spinocerebellar ataxia type 3 (SCA3). MJD is the most common dominantly inherited cerebellar ataxia in the world [30]. The neurological phenotype in MJD, which can range widely, has been classified into at least three subtypes with characteristic features: cerebellar ataxia with ophthalmoparesis and pyramidal tract signs (type II), with additional extrapyramidal signs (type I), or with distal muscular atrophy (type III) [31]. In early descriptions of MJD, muscle alterations were observed although studies of muscle involvement have been limited [31], [32], [33].

ATX3 is a highly conserved protein throughout evolution. We previously observed that the murine homolog (mATX3) exhibits ubiquitous expression, including during embryonic development [34]. Furthermore, mATX3 is highly expressed in muscle cells from embryonic development through adult ages, and its transcription is activated to a great extent in muscle-differentiated P19 cells [34]. To date, besides a potential involvement of ATX3 in transcriptional repression, DNA repair [35], and in endoplasmic reticulum associated protein degradation (ERAD) [28], no other cellular functions for ATX3 are known, and no physiological substrates for its DUB activity have been identified.

In this study we assessed the role of mATX3 in muscle differentiation. We found that mATX3 is strongly expressed in the early myotome of E9.5 mouse embryos, and it colocalizes with myosin in differentiated myogenic fibers. We found that mATX3 depletion in C2C12 cells perturbs the phenotypical response to differentiation induction, such as cell alignment and elongation, and also cellular proliferation. In addition, we observed that levels of α5 and α7 integrin subunits, as well as many other proteins implicated in the integrin signaling pathway, were altered in mATX3-depleted cells. Altogether, our data suggest that mATX3 is contributing to muscle differentiation by preventing the degradation of integrin subunits through the UPS.

## Results

### Ataxin-3 is highly expressed in the early differentiated myotome and in muscle fibers

To characterize the expression of mATX3 in the developing muscle, we performed double immunofluorescence labeling for mATX3 and myogenic markers in mouse embryo sections from E9.5 to E18.5.

**Figure 1:**
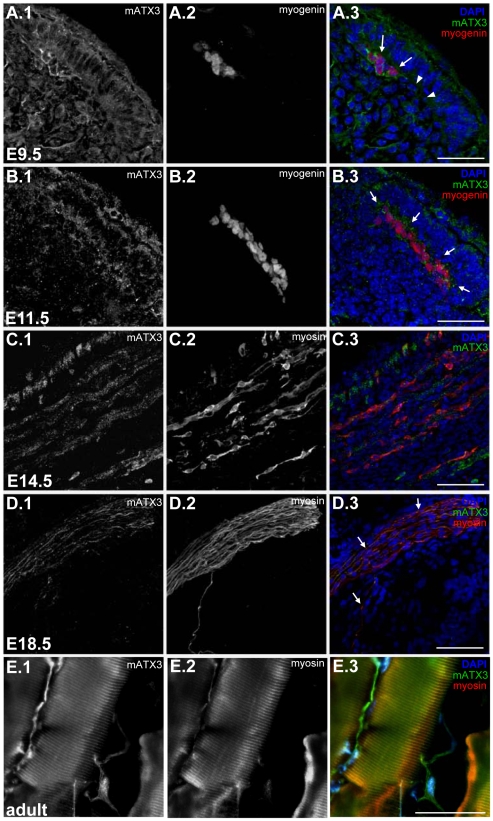
Mouse ataxin-3 (mATX3) is highly expressed in the early differentiated cells in the myotome,as well as in embryonic and adult muscle fibers. Maximum projection of confocal optical z-series from sagittal 15 µm sections of E9.5 (A), E11.5 (B), E14.5 (C) and E18.5 (D) mouse embryos labeled for mATX3 and the myogenic differentiation markers myogenin (A, B) or myosin (C, D). (A) mATX3 (green, arrows) is highly upregulated in differentiated myogenin-positive cells (red) when comparing with younger myogenin-negative cells in the myotome (arrowheads). (B) strong staining for mATX3 (green, arrows) remains present in E11.5 elongated myogenin-positive myocytes (red). (C) E14.5 intercostal muscle showing a fiber-like labeling pattern of mATX3 (green) colocalising with myosin-positive fibers (red). (D) mATX3 (green, arrows) remains strongly expressed at late fetal stages in myosin-positive fibers (red). (E) mATX3 (green) is expressed in adult myosin fibers (red). (1) mATX3 staining, (2) myosin (MHC) or myogenin labeling, (3) triple overlay of mATX3, myogenic marker, and DAPI. Scale bars represent 80 µm.

Although being broadly expressed, detected in the dermomyotome, and throughout the whole extent of the myotome, mATX3 is surprisingly up-regulated at E9.5 in elongated myogenin-positive cells (Figure 1A). This expression pattern is also observed at in the differentiated myotome at E11.5 (Figure 1B).

Immunoreactivity was also detected in muscle fibers analyzed later on (E14.5, E18.5, and adult) with no predominance in muscle type (Figure 1C, D, E).

The expression pattern suggests that mATX3 is present in all stages of myogenesis, with highest immunoreactivity in earlier stages of differentiation. Therefore, ATX3 may be implicated in muscle development.

### Ataxin-3 depletion perturbs the phenotype of differentiating C2C12 cells

**Figure 2:**
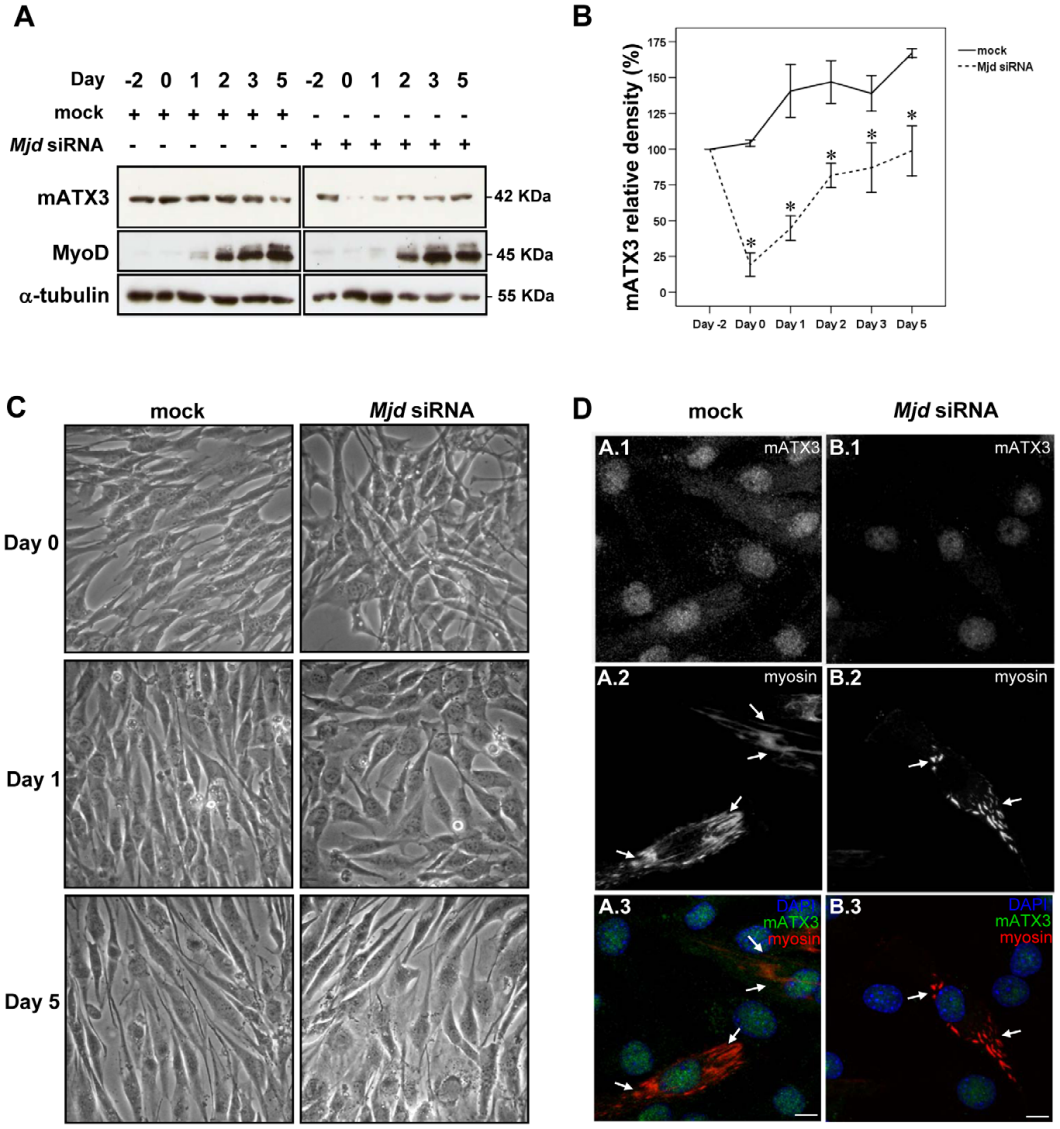
Expression profile of mATX3 during C2C12 differentiation and phenotype of mATX3-depleted cells by *Mjd* siRNA transfection. Cells were transfected at Day -2 and maintained in growth medium (GM) for 48 hrs (Day 0), then the medium was replaced by differentiation medium (DM) and changed every day until Day 5. (A) Whole cell extracts of undifferentiated (Day -2 and Day 0) and differentiated cells (Day 1 to Day 5) were analyzed for mATX3, MyoD and α-tubulin in mock and *Mjd* siRNA transfected cells. The amounts of ataxin-3 and MyoD were determined by immunoblotting. The myogenic factor MyoD is not a target of mATX3. Both mock and *Mjd* siRNA-transfected cells show that this MRF is upregulated upon differentiation. (B) Relative band density of mATX3 in each day (comparing with Day -2) shows that the *Mjd* siRNA has maximum efficiency two days after transfection (Day 0). The results were normalized for α-tubulin and correspond to the average of three independent transfections ± SEM (error bars). * statistical significance p<0.05 using Student t-test. (C) Phenotype of the *Mjd* siRNA C2C12 cells during differentiation. At Day 0 and Day 1, when mATX3 depletion is higher, the *Mjd* siRNA cells show extensive cell-cell misalignment, and rounder shape compared to mock-transfected cells. Although the mATX3 levels were partially restored at Day 5, the knocked-down cells still present some misalignment. (D) The maximum projection of confocal optical z-series images of mATX3 and myosin double-stained cells at differentiation Day 5. In contrast to mock control cells that show a well organized myosin cytoskeleton (arrows), mATX3-depleted cells show an abnormally fragmented myosin staining with a very short filament pattern. (1) mATX3 staining, (2) myosin (MHC) labeling, (3) triple overlay of mATX3, myogenic marker, and DAPI (DNA marker). Scale bars represent 10 µm.

To study the role of mATX3 in myogenic differentiation we transfected C2C12 myoblast cells with siRNA targeting the *Mjd* transcript, and induced their differentiation by switching from growth medium (GM) to a serum-limited differentiation medium (DM). The expression levels of mATX3 in *Mjd* siRNA-depleted cell extracts collected over time were analyzed by immunoblotting and compared with controls (mock transfection). The choice of the ideal time point to induce differentiation (Day 0) was based on the maximum suppression of mATX3 expression obtained two days after *Mjd* siRNA transfection (Day -2), with a reduction to about 18% of normal mATX3 levels (Figure 2A, B). In a pattern consistent with a putative role in myogenic differentiation, mATX3 levels in C2C12 control cells increase upon induction of differentiation at Day 0 (Figure 2A, B, mock).

After induction of differentiation (Day 0 to Day 5), *Mjd* siRNA-transfected cells were phenotypically different from mock-transfected cells: cell shape was rounder, with very thin cell extensions and extensive cell-cell misalignment (Figure 2C). At Day 5 the mATX3-depleted cells showed a very immature cytoskeleton, with myosin-containing filaments fragmented in the cytoplasm that failed to elongate as their control counterparts (Figure 2D). To exclude the possibility that the use of a dicer-generated pool of *Mjd* siRNAs might be interfering with other similar mRNAs, we analyzed the levels of the transcripts encoding the josephin-domain proteins (*Josd1* and *Josd2*). Quantitative real-time PCR revealed no significant differences for these transcripts between mock and *Mjd* siRNA transfected C2C12 cells at Days 0 and 1 of differentiation (Figure S1). These results suggest that the phenotype observed in C2C12 cells transfected with *Mjd* siRNA was specifically due to mATX3 knockdown, and that this protein is required during the differentiation process. Consistently, at Day 5, when mATX3 levels were restored to the levels of Day -2, the phenotype started to revert (Figure 2C).

To determine whether the mATX3-depletion phenotype was due to direct alteration of the myogenic transcriptional program, we analyzed the levels of the myogenic regulatory factors MyoD (Figure 2A) and myogenin (not shown) by immunoblotting. No differences were observed between *Mjd* siRNA and mock-transfected differentiated cells, suggesting that mATX3 does not perturb the onset of the myogenic program but has instead downstream targets.

### Ataxin-3 regulates α5 and α7 integrin subunit levels during myogenic differentiation

Providing a link between the extracellular matrix and the cell cytoskeleton, integrins play a critical role for the cytoskeletal modifications induced by myogenic differentiation. We sought to determine whether the abnormal phenotype of mATX3-depleted cells could be due to a perturbation on integrin levels and analyzed the expression pattern of two integrin subunits known to play a role in myogenesis: α5 integrin subunit and α7 integrin subunit.

**Figure 3:**
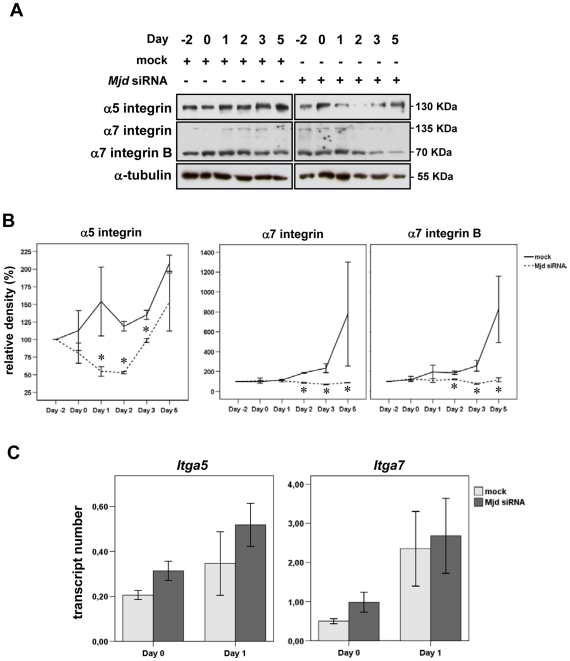
Ataxin-3 regulates the protein levels of α5 and α7 integrin subunits. (A) The amounts of α5 and α7 integrin subunits were determined by immunoblotting. Whole cell extracts of undifferentiated (Day -2 and Day 0) and differentiated cells (Day 1 to Day 5) were analyzed for α5 and α7 integrin subunits and α-tubulin in mock and *Mjd* siRNA transfected cells. (B) Relative band density of α5 integrin, α7 integrin (A+B) and α7B integrin subunits in each day (comparing with Day -2), and for each transfection condition. The results were normalized for α-tubulin and correspond to the average of three independent transfections ± SEM (error bars). (C) Ataxin-3 is not regulating the amounts of *Itga5* and *Itga7* transcripts. Mock and *Mjd* siRNA transfected C2C12 cells at Day 0 and Day 1 of differentiation, show identical transcript levels of *Itga5* and *Itga7* that were measured by real-time RT-PCR. The results were normalized for the *Hprt1* gene and correspond to the mean of three independent transfections ± SEM (error bars). * statistical significance p<0.05 using Student t-test.

**Figure 4:**
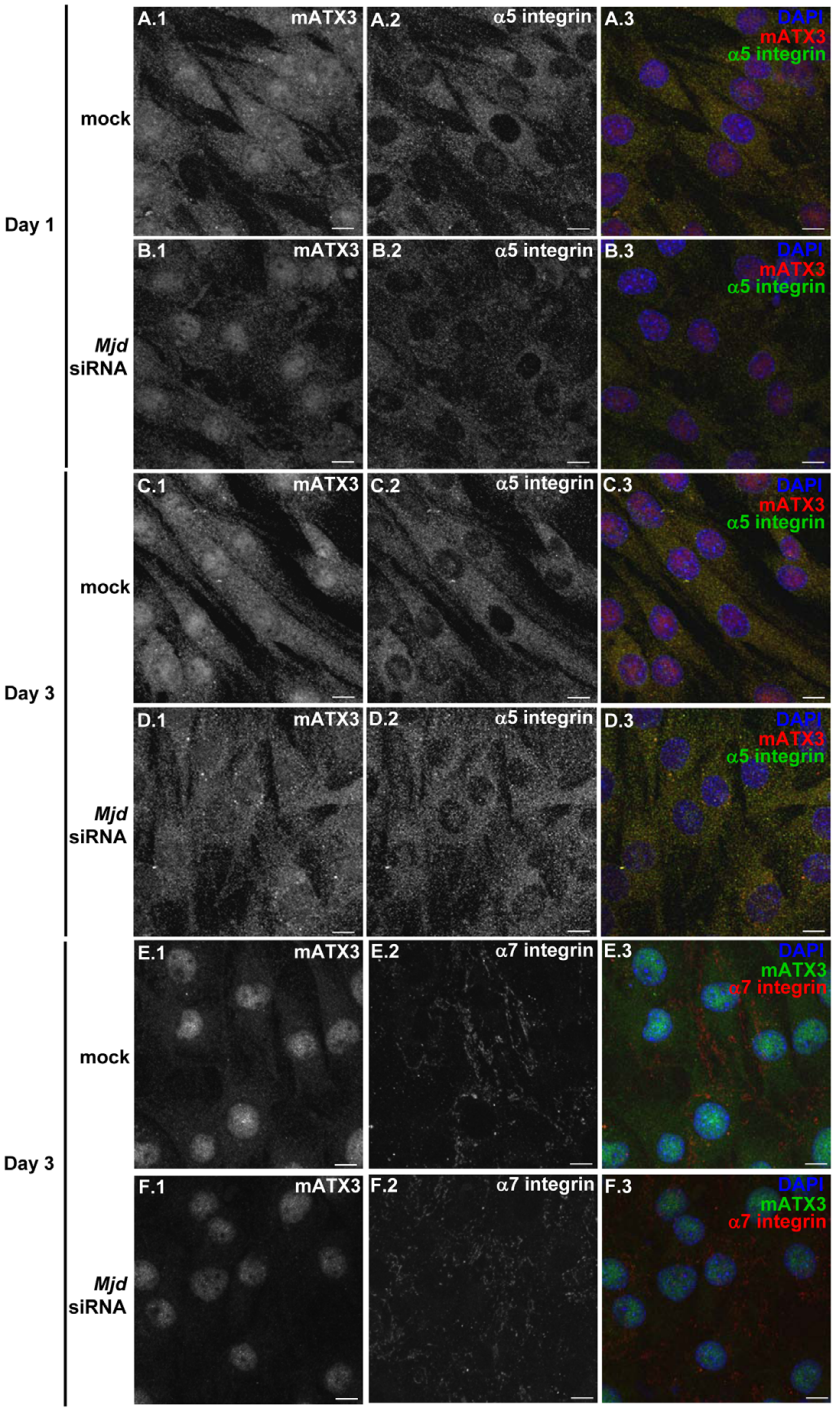
α5 and α7 integrin subunits are down-regulated in mATX3-depleted cells. Maximum projection of confocal z-series images of double immuno-stained cells for mATX3 (1) and α5 or α7 integrin subunit (2). (A–D) C2C12 differentiated cells labeled with mATX3 (red) and α5 integrin subunit (green): at Day 1, mATX3-depleted cells are misaligned and exhibit reduced levels of mATX3 and α5 integrin subunit (B), compared to controls (A); and at Day 3, the *Mjd* siRNA transfected cells still present lower amounts of mATX3 and α5 integrin subunit and considerable misalignment (D) in comparison to controls (C). Note that the stronger reactivity for mATX3 in *Mjd* siRNA cells at Day 3 (D.1 vs B.1) is accompanied by a similar increase in α5 integrin subunit reactivity (D.2 vs B.2,). (E–F) mATX3-depleted and differentiated cells at Day 3 labeled with mATX3 (green) and α7 integrin subunit (red) show reduced levels of mATX3 and α7 integrin subunit (F) compared to controls (E). DNA was stained with DAPI (blue) as shown in the overlay (3). Scale bars represent 10 µm.

Immunoblotting detection of α5, α7(A+B) and α7B integrin subunits in mock transfected C2C12 cells revealed that while the α5 subunit shows an immediate increase upon differentiation, and the α7 subunits increase more progressively (Figure 3A, B), which is in accordance with previously described expression patterns [36]. In contrast, we observed a decrease in the expression of both integrin subunits in *Mjd* siRNA-treated cells (Figure 3A, B). Interestingly, the α7 subunits levels were consistently maintained low until Day 5, while the α5 subunit levels decreased significantly from Day 0 to Day 2, and then started to increase at Day 3 until Day 5. This suggests that the α5 integrin subunit may be directly dependent on the levels of mATX3, registering a recovery as *Mjd* siRNA loses effect (see Figure 2 A, B). In contrast, the α7 integrin subunit levels were not able to recover during the experiment time course, suggesting that the relation with mATX3 may be indirect or that the effect of mATX3 depletion is less reversible in this case. The results obtained by immunoblotting were confirmed by co-immunostaining of mATX3 and α5 integrin subunit at Days 0 and 3 and mATX3 and α7 integrin subunit at Day 3 in *Mjd* siRNA-transfected C2C12 cells (Figure 4). Despite some increase of mATX3 levels at Day 3, comparing to Day 0 and Day 1, the *Mjd* siRNA cells still exhibited round shape, cell-cell misalignment and decreased amounts of α5 and α7 integrin subunits (Figure 4).

To define whether mATX3 affects the expression of α5 and α7 integrin subunits at the mRNA or protein level, we extracted total RNA from mock and *Mjd* siRNA-transfected undifferentiated (Day 0) and differentiated cells at Day 1 (the days of maximal depletion of *Mjd* transcript), and performed quantitative real-time RT-PCR to determine the amounts of the respective mRNAs (*Itga5* and *Itga7*). No significant differences between mock and siRNA-depleted cells were observed for *Itga5* or *Itga7* mRNA levels (Figure 3C), suggesting that the effect on α5 and α7 integrin subunit expression occurs at the protein level.

### Ataxin-3 interacts with α5 integrin subunit and represses its degradation

Several observations led us to consider the possibility that mATX3 might regulate the α5 integrin subunit degradation through the UPS: i) the expression profile of α5 integrin subunit mirrors that of mATX3 in mock- and *Mjd* siRNA-transfected C2C12 cells undergoing differentiation, ii) *Itga5* transcript levels remain unaltered in the mATX3-depleted cells, iii) ATX3 is a DUB, and iv) α5 integrin subunit levels are known to be regulated by the UPS [15]. Therefore, we first tested mATX3 for DUB activity. We expressed several recombinant proteins, and isolated the monomeric protein by gel filtration chromatography (Figure S2 and Materials and methods S1) [37]. Both the entire protein (mATX3), and the josephin domain (mATX3:jos) by itself, were able to cleave the fluorogenic substrate ubiquitin-AMC, and this activity was inhibited by mutation of the active site cysteine in mATX3:C14A and mATX:jos:C14A proteins (data not shown). As shown for other homologues, mATX3, specifically its josephin domain (mATX3:jos), is able to cleave either K48 or K63-linked polyubiquitin chains, the cysteine 14 residue being essential for this activity (Figure S2).

**Figure 5:**
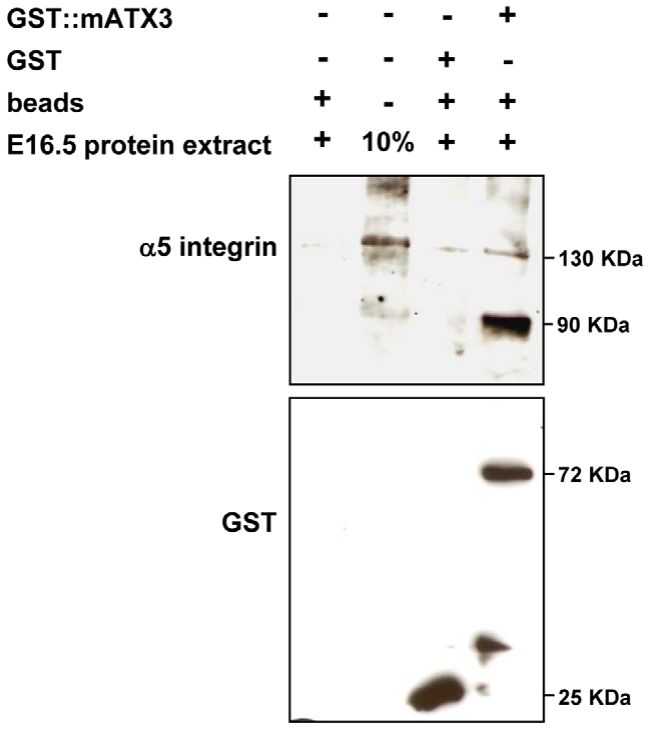
GST-tagged mATX3 pull-downs α5 integrin subunit from an embryo extract. Purified recombinant GST::mATX3 protein was incubated with a whole protein extract from an E16.5 mouse embryo. Recombinant GST alone was used as control. GST pulled down samples were analyzed by immunoblotting, first with the anti-α5 integrin antibody, and then with the anti-GST antibody, following stripping. Lane 2 corresponds to 10% of the input where α5 integrin subunit can be detected as with a band of 130 KDa. Lane 4 shows that GST-mATX3 was able to pull-down α5 integrin. Note that a band of 90 KDa was also detected, which might correspond to a cleaved product of α5 integrin generated during the assay. None of the α5 integrin bands were detected in the controls (lanes 1 and 3: beads alone or beads+GST, respectively).

Considering the possibility that α5 integrin subunit might be a substrate of mATX3, and that its deubiquitination could act to prevent its degradation through the UPS, we tested the interaction between these two proteins. In GST-pull down assays employing recombinant GST:mATX3 and total protein extracts from E16.5 mouse embryo, endogenous α5 integrin subunit co-precipitated with GST-mATX3 (Figure 5), confirming that a direct or indirect interaction between ATX3 and α5 integrin subunit occurs during mouse embryonic development. The 90KDa band pulled by GST:mATX3 and detected with the anti-α5 integrin antibody might correspond to a cleaved form of the protein.

**Figure 6:**
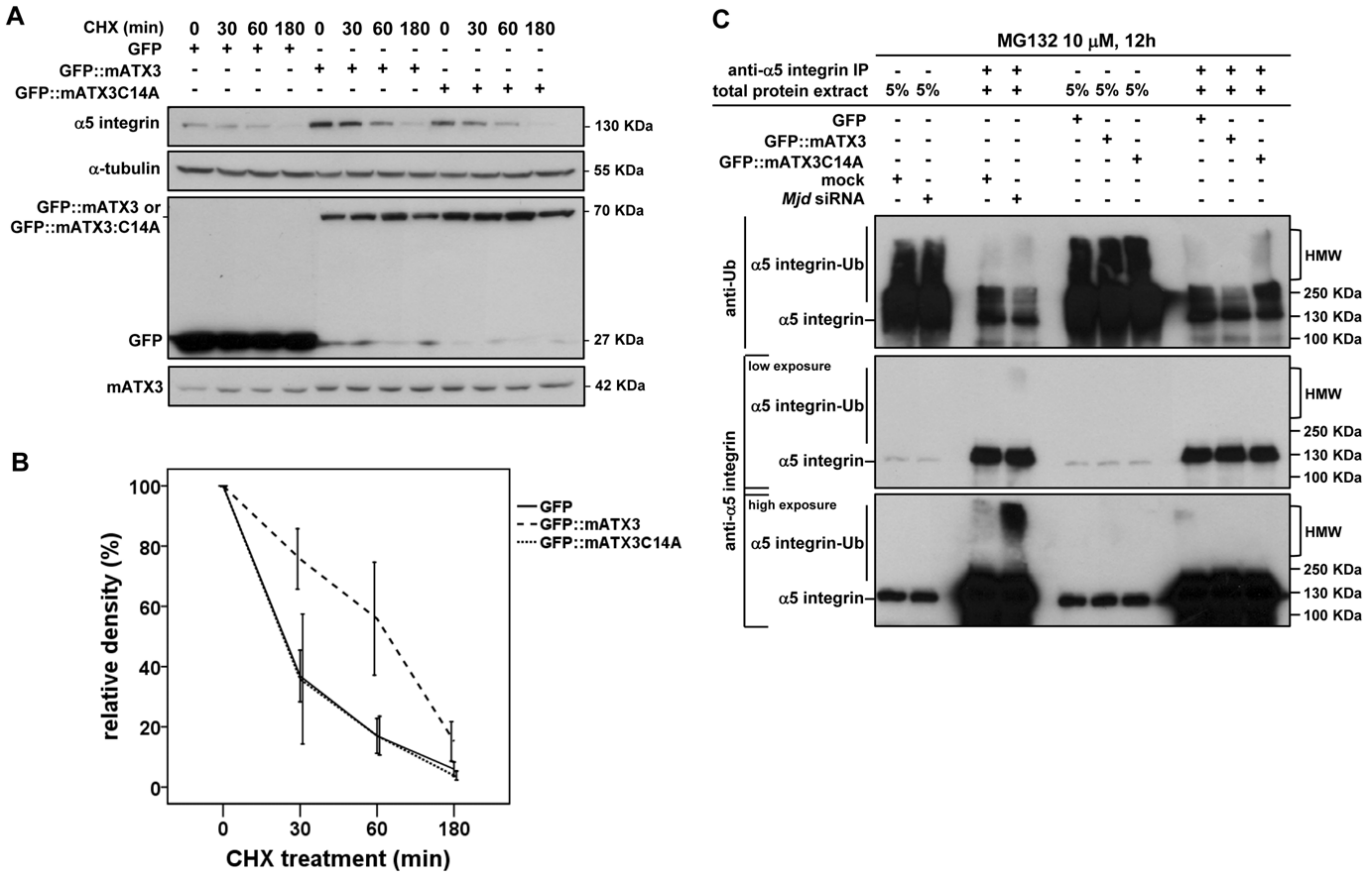
Ataxin-3 stabilizes α5 integrin subunit and represses its proteolysis. (A) Effect of mATX3 overexpression in the half-life of α5 integrin subunit. C2C12 cells were transfected with the pEGFP:Mjd (GFP::mATX3), the pEGFP:MjdC14A (GFP::mATX3C14A) or the pEGFP-C1 (GFP)-empty plasmids, and after 48 hrs cells were treated with cycloheximide for 0, 30, 60 or 180 min. Whole cell extracts were collected for these time points and analyzed for the levels of endogenous α5 integrin subunit by immunoblotting. The overexpression of GFP::mATX3, but not the of GFP or the catalytic mutant GFP::mATX3C14A, was shown to stabilize the levels of α5 integrin subunit. (B) The graph represents the relative amounts of α5 integrin subunit in GFP, GFP::mATX3 and GFP::mATX3C14A overexpressing cells at various cycloheximide treatment times. The levels of endogenous mATX3 were similar in all the transfections. The results were normalized for α-tubulin and correspond to the average of three independent transfections ± SEM (error bars). (C) Denature-renature immunoprecipitation of α5 integrin subunit from *Mjd* siRNA, mock, GFP, GFP::mATX3, or GFP::mATX3C14A transfected C2C12 cells treated with the proteasome inhibitor MG132 at 10 µM, during 12 h. The immunoprecipitated forms of α5 integrin subunit were separated in a SDS-PAGE gel and blotted for ubiquitin and α5 integrin subunit (low and high exposure of the same blot). An increase of ubiquitinated forms of α5 integrin subunit was detected in mATX3-depleted cells. Cells overexpressing GFP::mATX3 showed a reduction in ubiquitinated α5 integrin subunit forms. No decrease of these species was observed in cells overexpressing the catalytic mutant GFP::mATX3C14A.

To determine if mATX3 acts to inhibit α5 integrin subunit degradation during myogenesis, we assessed the levels of endogenous α5 integrin subunit upon protein synthesis inhibition with cycloheximide in C2C12 GFP:mATX3 overexpressing cells. The α5 integrin subunit was more abundant in these cells than in control GFP-transfected cells or in GFP::mATX3C14 (DUB catalytic mutant) overexpressing cells (all presenting the same levels of endogenous mATX3) (Figure 6A, B), indicating that mATX3 is able to stabilize this protein and prolong its half-life. Indeed, we verified by denature-renature immunoprecipitation assays an increase of high molecular weight (HMW) ubiquitinated α5 integrin subunit species upon UPS inhibition with MG132 in mATX3-depleted cells (Figure 6C), which implies that mATX3 is repressing its proteolysis at the UPS level. In contrast, cells overexpressing mATX3 show decreased levels of ubiquitinated α5 integrin subunit species, but not cells overexpressing the catalytic mutant mATX3C14A that showed a similar pattern to the empty vector-transfected cells (Figure 6C).

### Proteomic analysis of ataxin-3 depleted-cells leads to changes in the integrin signaling pathway

Aiming to identify proteins required for muscle differentiation, the expression of which is altered upon mATX3 repression, we performed a large-scale proteomic analysis of undifferentiated *Mjd* siRNA-treated C2C12 cells (Day 0). Two independent samples of total proteins extracted from C2C12 cells (mock and *Mjd* siRNA transfections) were labelled using the iTRAQ technology, separated by isoelectric focusing, and analyzed by LC-ESI-MS/MS. The average number of proteins detected per sample was 1634 proteins. We considered proteins with altered expression levels to be those presenting changes of 20% or more of the normal levels (1.2<ratio<0.8) and significant p-value. At Day 0, when the levels of mATX3 were maximally reduced in *Mjd* siRNA transfected cells, a total of 106 proteins were found to be altered (Table S1). Using the Ingenuity Pathways Analysis® software, these proteins were grouped into six functional networks (according to the software annotation) (Table S1). In agreement with the phenotype observed, two of these networks were “Skeletal and muscular system development and function/Tissue morphology/Cancer” and “Cellular development/Embryonic development/Cellular assembly and organization”.

**Figure 7:**
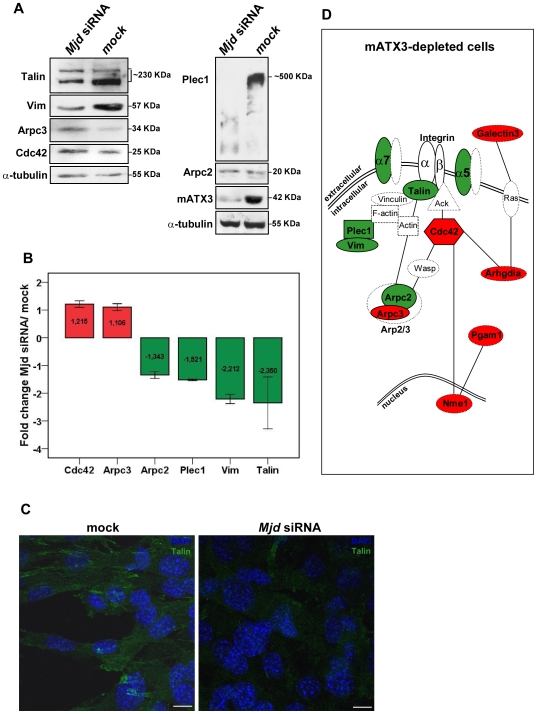
Ataxin-3 depletion affects the levels of proteins involved in the integrin signaling cascade which regulates cytoskeleton organization, cell adhesion and motility. (A) The levels of Cdc42, Arpc2, Arpc3, Plec1 and Vim, were found to be altered in a proteomic analysis of mATX3-depleted C2C12 cells (Day 0). These results were confirmed by immunoblotting of whole cell extracts of undifferentiated (Day 0) mock and *Mjd* siRNA transfected cells. The levels of Talin were also found to be downregulated by immunoblotting analysis. (B) Fold change correspondent of relative band density of each protein in *Mjd* siRNA transfected cells comparing with control cells. The results were normalized for α-tubulin and correspond to the average of three independent transfections ± SEM (error bars). (C) Maximum projections of confocal optical z-series images of immunofluorescence staining for Talin (green) of undifferentiated mock or *Mjd* siRNA-transfected C2C12 cells (Day 0). DNA was stained with DAPI (blue). Scale bars represent 10 µm. (D) Scheme of the proteins showing altered levels upon mATX3-depletion in C2C12 cells and related with the integrin signaling pathway (based in the analysis performed with the Ingenuity Pathway Analysis® software). Upregulated and downregulated proteins found in the proteomic analysis, as well as α5 and α7 integrin subunits, are shown in red and green, respectively. Proteins with confirmed altered levels by further immunoblotting and/or immunofluorescence are represented by full-surrounded lines, and the ones only identified as altered in the proteomic analysis by dashed-surrounded lines.

To identify the effect of mATX3 depletion in the integrin signaling pathway we overlaid the proteins altered in this condition with the integrin signaling canonical pathway using the above mentioned software. Interestingly, three proteins involved in the integrin signal transduction cascade that specifically leads to cytoskeleton rearrangements and cell motility showed altered levels in the mATX3-depleted cells: two subunits of the actin related protein 2/3 (Arp2/3) complex, Arpc2 (−1.3-fold) and Arpc3 (+1.3-fold); and the GTPase Cdc42, which, in turn, activates the Arp2/3 complex (+1.3-fold) (Table S1). In addition, two structural components of the cytoskeleton, plectin 1 (Plec1) and vimentin (Vim), were down-regulated, −1.3 and −1.3-fold, respectively (Table S1). The altered levels of Arpc2, Arpc3, Cdc42, Plec1 and Vim in the mATX3-depleted cells were confirmed by immunoblotting (Figure 7A, B). In fact, the fold change values of these proteins found in the proteomic analysis and in the immunoblotting were very similar, except for Vim that showed a higher fold decrease in the immunoblotting detection (Figure 7B and Table S1). Furthermore, the levels of Talin (a cytoskeleton protein that directly interacts with the intracellular domain of integrins and participates in actin filament assembly, and in cell spreading/migration) showed to be decreased in *Mjd* siRNA-treated cells by both immunoblotting and immunofluorescence assays (Figure 7A, B, C). Alterations in the amounts of these proteins (Figure 7D) suggest that the phenotypic alterations observed in mATX3-depleted C2C12 cells might reflect a dysregulation of specific integrin-mediated signaling cascades leading to changes in cytoskeleton, cell adhesion and motility.

## Discussion

Myogenesis is one of the best studied systems of cell differentiation. The muscle, in particular the striated muscle, is well known by its unique structure consisting of a very well organized cytoskeleton that enables its characteristic contractile function. However, the formation and maintenance of these structures, as well as the entire event of myogenic differentiation from MPCs into the final myofibers, are very dynamic processes and require a high coordination between the synthesis, degradation and assembly of key proteins. Degradation of muscle proteins occurs through several mechanisms but more importantly by the UPS. The fast and time-specific regulation of protein levels by the UPS, is crucial for muscle development [8]. Several key factors in myogenesis are known to be regulated by the UPS, namely myogenic factors, such as MyoD and Myogenin, and membrane proteins, like β-catenin and integrins [8], [10], [13], [14], [15].

The human ATX3 protein possesses DUB activity, suggestive of a role in the UPS [21], [22], [23], [24], [38]. In this study, we further confirmed the orthology of the mouse *Mjd* gene by demonstrating that mATX3 protein possesses DUB activity, similarly to other ATX3 and josephin proteins [39], [40]. mATX3 and other DUBs, such as Usp22 [41], Usp2-45 and Usp2-69 [42] are expressed during mouse embryonic development but little is known about their physiological function in that process. Here, we were able to show that ATX3 plays a role during muscle differentiation. Mouse ATX3 is highly expressed in heart (not shown) and skeletal muscle, both in the precursor structures (epithelial somite, dermomyotome and early myotome) and in myogenic fibers, indicating that this DUB might be involved in all stages of muscle development.

In this study, we focused on the involvement of mATX3 in the early stages of myogenic differentiation. *Mjd* siRNA depletion in C2C12 cells was shown to induce morphological alterations (round shape), defects in cell-cell alignment, and fragmented myosin-positive filements. This phenotype was specifically due to mATX3 repression as the levels of other josephin domain-containing homologue genes remained unchanged in *Mjd* siRNA cells. Furthermore, no alterations in the amounts of MyoD were found during the differentiation process, indicating that mATX3 is not affecting the myogenesis pathway onset.

We found that the phenotype observed in the mATX3-depleted cells is likely due to a decrease in the levels of both α5 and α7 integrin subunits, known to be important for proper muscle differentiation. During muscle differentiation, integrins play fundamental roles and are strictly regulated, presenting highly specific expression patterns for each discrete phase of myogenesis [43], [44]. Mouse ATX3 was found to regulate the amount of these two integrin subunits at the protein level given that their mRNA levels remain unaltered in the mATX3-knock down cells. The fact that the α7 integrin subunit levels remain low at late differentiation stages, even when the amount of mATX3 was partially restored, suggests that mATX3 might have an indirect effect over this integrin subunit, probably affecting (in an irreversible manner) the degradation of other proteins that control its up-regulation or maintenance. In contrast, α5 integrin subunit levels paralleled those of mATX3 in the *Mjd* siRNA-treated cells suggesting a more direct regulation. Consistently with this biological interaction, the α5β1 integrin and mATX3 *in vivo* expression patterns seem to correlate in the myotome of E9.5 and E11.5 mouse embryos and in C2C12 cells [34], [43], [45]. In this work, we have also shown that: (1) mATX3 interacts with the α5 integrin subunit; (2) overexpression of mATX3 stabilizes α5 integrin subunit and increases its half-life; and (3) the amount of ubiquitinated α5 integrin subunit increases in mATX3-depleted cells when the proteasome is inhibited. Our results, together with the fact that α5 integrin subunit is degraded by the UPS (Kaabeche et al. 2005), lead us to suggest that this integrin subunit might be a substrate of mATX3 DUB activity, which would act to prevent its UPS-mediated degradation. Decreased levels of α5 integrin subunit in mATX3-depleted cells might cause an alteration in the cell surface molecules, and consequently an impairment of cell-cell and cell-matrix interactions that might, in turn, lead to the observed phenotypic changes of the mATX3-depleted C2C12 cells.

The proteomic profile of mATX3-depleted undifferentiated C2C12 cells pointed to an involvement of the integrin signaling transduction cascades.Three downstream targets implicated in the actin cytoskeleton assembly and organization presented altered levels: Cdc42, Arpc2 and Arpc3 (subunits of the Arp2/3 complex). In mATX3-depleted cells, Arpc2, one of the core subunits of the Arp2/3 complex, is down-regulated; this can lead to an inappropriate assembly of this nucleation complex. The decrease is Arpc2 is possibly due to a decrease of the integrin core signaling, specifically of the α5 and α7 integrin subunit-containing receptors. On the other hand, Arpc3 (Arp2/3 lateral subunit), as well as Cdc42 (Rho-GTPase that activates Arp2/3-mediated actin polymerization [46]) were upregulated. This could be a result of the compensation by other types of integrin receptors, in an attempt to overcome the dysregulation of the actin cytoskeleton.

Additionally, two other cytoskeleton-associated proteins, were shown to be down-regulated: plectin1 (Plec1) and vimentin (Vim). Interestingly, α6β4 integrin binds to Plec1 preventing its binding to F-actin but not interfering with intermediate filament binding [47]. In addition, vimentin also binds to Plec 1 in intermediate filaments [48]. These alterations suggest that the cytoskeleton structures in general may be deregulated in the absence of mATX3, which may explain the altered morphologic features of mATX3-depleted cells.

Cdc42 upregulation may also be due to the higher levels of ARHGDIA, a Rho GDP dissociation inhibitor that is also an ATX3 interactor [49], and is known to increase the translocation of Cdc42 from membrane to cytosol [50]. The upregulation of galectin-3 (Lgals3) is consistent with the high levels of Cdc42 since the former is responsible for its activation via Cspg4 [51]. This upregulation of Lgals3 may be an attempt to trigger other integrin receptors in order to overcome α5β1 and α7β1 down-regulation at the cell surface. Furthermore, the down-regulation of talins (actin and integrin binding proteins) may explain *per se* the misalignment and apparent failure of adhesion observed in the mATX3-depleted cells given their key role in focal adhesions and cell spreading [52].

Taking all the evidence presented, ATX3 seems to be crucial for the correct cell-cell interaction and alignment, required for cell fusion and progression of myogenesis. However, in the recently reported mATX3 knockout mouse [53], muscle malformations were not described, suggesting that the phenotype presented by the *Mjd* siRNA C2C12 cells (an isolated system) during particular phases of myogenesis, might be overcome in a whole organism, such as the mouse embryo. This could be possible by the upregulation of partially redundant proteins, such as other mouse josephins. Compensation phenomena were also suggested to explain the lack of stronger phenotypes in some integrin knockouts [54], and redundancy of myogenic regulatory factors is well known in many animal models [55], [56], [57]. Nevertheless, specific muscle studies in this knockout mouse model are still required to exclude a muscle associated phenotype. Interestingly, recent work has shown that at higher temperatures the *C. elegans* ataxin-3 knockout animals have a severe motility defect, potentially correlated with some muscle deficits [29].

### Final remarks

Since the cloning of the MJD gene in 1994 [58], several cellular and animal models of MJD have been developed but, until now, its pathogenic mechanism remains unknown. Given that MJD is a dominant disease, a gain of function of mutant ATX3 was proposed as the cause of the pathogenesis. However, the knowledge of the normal function(s) of ATX3 may also be important for the unveiling of the pathogenic mechanism(s). Thus, we could consider that in MJD patients sequestration of normal ATX3 into the aggregates might lead to decreased levels of α5β1 and α7β1 integrin levels and, thereby, to an impairment of myogenesis either during development or in adult MSCs, that differentiate and give rise to new muscle fibers upon muscle injury, possibly leading to the muscle atrophy seen in some type III MJD patients [31], [32]. More importantly, the α5β1 integrin, as a critical regulator of the actin cytoskeleton, was shown to be very important in the regulation of spine morphogenesis and synapse formation in hippocampal neurons, presenting in this way a fundamental role in synaptic plasticity [59]. Since an impairment of synaptic transmission was reported in MJD transgenic *C. elegans* [60], it will be interesting to know if integrins are playing a role in this component of pathogenesis.

In summary, we report the physiological implication of the DUB mATX3 in myogenesis through the regulation of the integrin signaling transduction pathway. This is the first report of a direct target of the mouse ataxin-3 DUB activity, and also the first report of the involvement of a DUB in a developmental process. This novel biological role of ataxin-3 opens the possibility that ataxin-3 might play a similar function in neuronal differentiation and could also be important for the understanding of the pathogenic mechanism underlying MJD.

## Materials and Methods

### Antibodies

The primary antibodies used were: rabbit anti-ATX3 antiserum (kindly provided by H. Paulson), monoclonal antibody for ubiquitinylated proteins (FK2, Biomol International LP), rabbit anti-ubiquitin (Dako), anti-MHC (F59, DSHB), anti-myogenin (F5D, DSHB), rabbit anti-MyoD (provided by J. Harris), rabbit anti-α5 integrin subunit (Chemicon), goat anti-α5 integrin subunit (Santa Cruz Biotechnology (SCB)), rat anti-α7 integrin subunit (provided by A. Sutherland), anti-α-tubulin (clone AA4.3, DSHB), goat anti-GST (GE Healthcare), rabbit anti-GFP (SCB), anti-talin (C20, SCB), anti-Cdc42 (B8, SCB), anti-Arpc2 (C13, SCB), anti-Arpc3 (H17, SCB), anti-plectin (10F6, SCB) and anti-vimentin (DSHB). The peroxidase-conjugated secondary antibodies used for immunoblotting were: goat anti-mouse (Santa Cruz Biotechnology), goat anti-rabbit (Santa Cruz Biotechnology), goat anti-rat (Serotec), and donkey anti-goat (Santa Cruz Biotechnology). For double immunofluorescence assays, the Alexa Fluor (AF) secondary antibodies (Molecular Probes) used were the following: AF 488 goat anti-rabbit, AF 568 goat anti-mouse, AF 594 goat anti-rat, AF 594 goat anti-rabbit, and AF488 donkey anti-rabbit.

### Embryo collection and histology

All animal procedures were conducted in accordance with European regulations (European Union Directive 86/609/EEC). Animal facilities and the people involved in animal experiments (MCC, FB) were certified by the Portuguese regulatory entity - Direcção Geral de Veterinária. All the protocols performed were approved by the joint Animal Ethics Committee of the Life and Health Sciences Research Institute, University of Minho. For embryonic expression studies, the day of detection of a vaginal plug was designated as embryonic day (E) 0.5. Pregnant C57Bl/6 (Harlan Interfauna Iberica, SA) females were sacrificed by CO_2_ inhalation, and embryos from E9.5, E11.5, E14.5, E15.5 and E18.5 stages were collected in cold PBS. The embryos were fixed as previously described [43]. Embryos were cryosectioned in a Leica CM1900 cryostat into 15 µm- thick sagittal slices, which were collected on Super Frost slides and stored at −20°C.

### RNA interference

Dicer small interference RNA (siRNA) was prepared using the Block-iT™ RNAi Topo transcription kit (Invitrogen) for topo-mediated generation of templates and production of double-stranded RNA (dsRNA), and the Block-It™ Dicer RNAi kit (Invitrogen) for the generation, purification of Mjd-specific d-siRNA, using the manufacturer's conditions. Briefly, we started to generate an *Mjd* cDNA fragment by PCR, consisting in the first (5′-3′) 646 bp, using primers mmMJD106/mmMJD10, the pMjd1 plasmid as template, and the Platinum Taq DNA Polymerase High Fidelity; this PCR product was processed using the abovementioned kits to produce the *Mjd* d-siRNA.

### C2C12 culture and transfection

Mouse C2C12 myoblasts were cultured in growth medium (GM) containing Dulbecco's modified Eagle's (DMEM) Glutamax (Gibco BRL), 20% fetal bovine serum (FBS) and 1% penicillin/streptomycin, in a 5% CO_2_ humidified chamber at 37°C. To induce myogenic differentiation, C2C12 cells at 80–100% confluence in GM where washed with phosphate-buffered saline (PBS) and grown in differentiation medium (DM) containing DMEM glutamax, 2% horse serum and 1% penicillin/streptomycin, that was changed every day. *Mjd* siRNA and mATX3 overexpression experiments consisted in transfecting of 1×10^5^ C2C12 myoblasts per well (6-well plates) with either 300 ng of *Mjd* siRNA and 2 µL of Lipofectamine 2000 (Invitrogen), or 2 µg of pEGFP:Mjd or pEGFP:MjdC14A and 5 µL of Lipofectamine 2000 in antibiotic-free medium (Opti-MEM, Life Technologies) for 6 hrs, after which the medium was changed for GM. Mock transfections were also performed in the same way but without siRNA, as well as transfections with the empty pEGFP-C1 vector (Clontech). At least, three independent experiments were performed. Regarding C2C12 differentiation, the day of transfection was named Day -2 of differentiation. Two days after (Day 0) the GM was changed for DM that was changed every day. Cellular extracts were collected from Days −2, 0, 1, 2, 3, and 5 of differentiation.

### Immunofluorescence staining

Slides containing the embryo slices from the several collected stages were placed in a humified chamber and the tissues were permeabilized in 0.2% Triton-X-100/PBS during 20 min, and blocked in 10% goat serum during 30 min. For double staining analysis, the slices were then incubated with the rabbit anti-ATX3 antiserum (1∶500) in conjunction with another primary antibody (all diluted in 1% BSA/PBS) overnight at 4°C. For immunofluorescence labeling, C2C12 cells were transfected after being seeded in coverslips. At the differentiation days 0, 1, 3, and 5, cells were washed briefly with PBS and fixed with 1% PFA/PBS, through 10 min, and permeabilized in 0.5% Triton-X-100/PBS (5 min). After blocking in 2% BSA/PBS (30 min), coverslips were incubated with the anti-ATX3 antiserum (1∶500) in combination with other primary antibody (all diluted in 2% BSA/PBS) overnight, at 4°C. Primary antibodies used were mouse anti-MHC (1∶20), mouse anti-myogenin (1∶20), goat anti-α5 integrin subunit (1∶100), rat anti-α7 integrin subunit (1∶100), and goat anti-talin (1∶100).Subsequently, slices and coverslips were washed with PBS and incubated for 1 h with the two corresponding Alexa Fluor (AF) secondary antibodies, diluted 1∶1000 in 1% BSA/PBS. After that, slices were stained with 4′,6-Diamidino-2-phenylindole dihydrochloride (DAPI, Sigma) diluted 1∶2000 in PBS during 5 min, and mounted with Vectashield (Vector Laboratories).

### Imaging

Embryo sections and C2C12 cells processed for immunofluorescence were photographed in an OLYMPUS IX 81 confocal microscope using the software OLYMPUS Fluoview1000 (FV viewer v.1.6.a). Optical z-series for both embryos and C2C12 cells were used to obtain maximum projections. C2C12 cells were photographed with a SONY digital camera coupled to a phase contrast Axiovert 200 Zeiss microscope, during differentiation after mock and *Mjd* siRNA transfections.

### Quantitative real-time RT-PCR

C2C12 total RNA (2 µg) from differentiation Day 0 and Day 1 (mock, and *Mjd* siRNA transfections) was reverse transcribed using the SuperScript First-Strand Synthesis System for RT_PCR (Invitrogen) with an oligo(dT) primer. Gene amount was assessed by quantitative real-time RT-PCR, using the QuantiTec SYBR Green PCR kit (Qiagen), 2 µL of total cDNA, and 500 nM of each primer. The pair of primers used to amplify the genes *Itga5*, *Itga7*, *Josd1*, *Josd2*, and *Hprt1* was respectively: Itga5(1)/Itga5(2) (148 bp); Itga7(1)/Itga7(2) (151 bp); Josd1(1)/Josd1(2) (116 bp); Josd2(1)/Josd2(2) (147 bp); and Hprt(3)/Hprt(4) (249 bp) (Table S2). The reactions were carried out in a real-time cycler LightCycler (Roche) using standard cyclic conditions indicated in the abovementioned kit.

### Protein synthesis inhibition

C2C12 cells were transfected with pEGFP:Mjd, pEGFP:MjdC14A or pEGP-C1 plasmids as abovementioned. Forty eight hrs after transfection, the medium was changed for DM. After 24 hrs, cells were treated with cycloheximide (Merck) at 50 µg/mL during 30, 60, or 180 min. Whole protein extracts were prepared from each experiment that was performed in triplicate.

### Preparation of total cellular and tissue extracts

For total protein extraction, C2C12 cells from the differentiation days − Day -2, Day 0, Day 1, Day 2, Day 3 and Day 5 − were washed briefly with PBS and collected in 500 µL of cell lysis buffer (1% Nonidet P-40, Complete (Roche Diagnostics) in PBS). Total protein extract from a mouse E16.5 embryo was collected after homogenization in 1 mL of tissue lysis buffer (0,25% Triton X-100, Complete (Roche Diagnostics) in PBS), sonication, and centrifugation. The total protein amount was quantified using the Bradford reagent (Sigma Aldrich) and appropriate dilutions were stored at −20°C. For total RNA extraction, cells from the differentiation Day 0 and Day 1 were washed with PBS and after centrifugation the cell pellet was stored at −80°C. Total RNA was purified using the Versagene kit (Gentra Systems) according to the manufacturer instructions.

### GST pull-down and immunoprecipitation

For GST pull-down assay, the purified GST or GST-tagged proteins (2 µg) (Materials and methods S1) were incubated rotating with 40 µL of glutathione-sepharose™ 4B beads (GE Healthcare), in a final volume of PBS/protease inhibitors, during 1 h, at 4°C. Whole E15.5 embryo proteins extract (150 µg) was added and incubated for 2 hrs, at 4°C, in a rotator. Beads were washed four times with PBS and bound proteins were eluted in 65 µL of Laemmli sample buffer, at 95°C, for 5 min. Itga5 was immunoprecipitated from cells using denature/renature immunopurification as previously described [24]. Briefly,5 µg of the anti-α5 integrin subunit antibody (Chemicon), previously cross-linked with protein G beads (Sigma),were incubated overnight, at 4°C with 500 µg of total protein extracts of C2C12 cells from Day 1 of differentiation subjected to 10 µM MG132 for 12 h.

### Proteomics

#### iTRAQ labeling and trypsin digestion

Total protein extracts were obtained from C2C12 cells from differentiation Day 0 (mock, and *Mjd* siRNA transfections). After quantification, 100 µg of total proteins from each condition was precipitated using six volumes of cold acetone to remove interfering substances. The samples were incubated at −20°C for 1 h and then centrifuged at maximum speed at 4°C. Protein pellet was ressuspended in 40 µL of 500 mM triethyl ammonium bicarbonate with 1% SDS, vortexed, and briefly sonicated until fully dissolved. The proteins were reduced using 2 µL of 50 mM tris-(2-carboxyethyl) phosphine for 1 h at 60°C, and cysteines were blocked with 200 mM methyl methane thiosulfonate (MMTS) for 10 min at room temperature. Ten micrograms of Sequencing Grade Modified Trypsin (Promega) diluted in water was added to each sample and incubated overnight at 37°C. The iTRAQ reagents (Applied Biosystems) were reconstituted in 70% ethanol, transferred to the respective sample and allowed to incubate for 1 h. Reagent 114 was added to the Day 0 mock, and 115 to Day 0 *Mjd* siRNA; and the labeled peptides were then combined and evaporated to dryness in a SpeedVac. Each sample was re-dissolved in 50 µL of 0,1% trifluoroacetic acid (TFA) and desalted using C_18_ empore disks.

#### Isoelectric focusing

Desalted samples were applied on a 13 cm IPG strip, pH 3–10 (GE Healthcare), rehydrated for 12 hrs and then focused in a IPGphor using the following parameters: hold at 500 V 1 h, linear gradient from 500–1000 V 15 min, hold at 1000 V 1 h, linear gradient from 1000 V–8000 V 30 min, hold at 8000 V 2 hrs. The strips were cut into 12 fractions and focused peptides were extracted from the gel using a TFA gradient. The sample were evaporated and desalted as mentioned above. Peptides were dissolved in 1% HCOOH just before LC-ESI-MS/MS analyses.

#### LC-ESI-MS/MS analyses

Around 6–8 µg of peptides from each fraction were analyzed using a nanoflow LC (Famos, Switchos, and Ultimate^Plus^; LC Packings - Dionex Corporation, Sunnyvale, CA, USA) coupled to a QSTAR Pulsar i ESI-hybrid Q-TOF tandem mass spectrometer (Applied Biosystems/MDS Sciex, Toronto, Canada). Peptides were concentrated and desalted on a precolumn (0.3X5mm C_18_ PepMap100, LC Packings), and eluted at 200 nL/min by increasing concentration of acetonitrile onto a self-packed C_18_ reverse phase column (75 µmX15 cm, Magic 5 µm 100 Å C_18_, Michrom BioResources Inc.) A linear 90 min gradient from 98% solvent A (97.8% water, 2% acetonitrile, and 0.2% formic acid) to 35% solvent B (95% acetonitrile, 4.8% water, and 0.2% formic acid) was used. Data from LC-MS/MS runs were converted to peak list files with the Analyst QS software (version 1.1).

#### Data analysis and protein identification

MS/MS spectra generated was analyzed using Protein Pilot (version 2.0, Applied Biosystems/MDS Sciex) search engine running over a *Mus musculus* database downloaded from NCBI. The default search settings used for quantitative analysis and protein identification were: trypsin cleavage with fixed MMTS modification of cysteine, iTRAQ labeling and variable methionine oxidation. We have considered only protein identifications with >95% statistical confidence in Protein Pilot. The quantification of the identified proteins was reported using the 114 (Day 0 mock) tag as the control with bias correction. For data analysis, a P-value<0.05 was considered for 115/114 comparison, and the expression ratio cut-off used was of 20%, i.e. were considered the ratio values smaller than 0.8 and higher than 1.2. Data analysis and molecular network pathways were created using the trial version of the Ingenuity Pathways Analysis Inc. 5.0 software, which in turn uses all the information known for a specific gene/protein including the one from their homologues in other species. The ratios were converted to fold change values through the negative inverse (-1/x) for values between 0 and 1, not affecting the values greater than 1.

### Immunoblotting

Total proteins from the several C2C12 differentiation days (20 µg) were resolved in appropriate SDS-PAGE polyacrylamide gels and then transferred to a nitrocellulose membrane (Hybond-C, GE Healthcare). Membranes were incubated, overnight at 4°C, with primary antibodies: anti-ATX3 antiserum (1∶5000), anti-myogenin (1∶50), anti-MyoD (1∶1000), rabbit anti-α5 integrin subunit (1∶5000), anti-α7 integrin subunit (1∶2000), anti-Arpc2 (1∶1000), anti-Arpc3 (1∶1000), anti-Cdc42 (1∶1000), anti-plectin (1∶1000), anti-talin (1∶1000) or anti-vimentin (1∶1000). All membranes were incubated with anti-α-tubulin (1∶500) for loading control. For the GST pull-down assays, bound proteins were resolved in an 8% SDS-PAGE polyacrylamide gel and transferred in the same abovementioned way. Blots were incubated with anti-GST (1∶7500) or rabbit anti-α5 integrin subunit (1∶5000) overnight at 4°C. The de-ubiquitination assays of the K48 and K63- multiubiquitin chains were immediately loaded in a 10% SDS-PAGE polyacrylamide gel, and transferred in the same above way. Membranes were incubated with a 1∶1500 dilution of a monoclonal antibody to ubiquitinylated proteins. For the immunoprecipitation assay, bound proteins were separated in a regular SDS-PAGE gel, and membranes were probed first with the anti-ubiquitin antibody (Dako) (1∶1000), and then with the rabbit anti-α5 integrin subunit antibody (1∶2000). Detection of the immunocomplexes was performed using the peroxidase-conjugated secondary antibodies anti-mouse (1∶10000), anti-rabbit (1∶10000), anti-rat (1∶10000), or anti-goat (1∶30000), and a SuperSignal West Pico Chemiluminescent Substrate (Pierce). The immunocomplexes signal was registered in ECL-films (Hyperfilm, GE Healthcare), and after that quantified using the software Quantity One 4.6.3 (Bio-Rad).

### Statistical analysis

The relative mean band density for each analyzed protein, in at least three independent immunoblottings, was compared between two groups of experiments (mock- and siRNA- transfected cells) using the t-test (calculated using the SPSS version 16.0 package). The same approach was used to analyze real-time PCR data. A significant value of *p*<0.05 was considered.

## Supporting Information

Materials and Methods S1 (0.02 MB PDF)

Figure S1 The transcript levels of the genes encoding other josephin-domain containing proteins *Josd1*, and *Josd2* for mock and *Mjd* siRNA transfected C2C12 cells at Day 0 and Day 1 of differentiation, were measured by quantitative real-time RT-PCR, and showed to be similar. The results were normalised for the *Hprt1* gene and correspond to the mean of three independent transfections +/− SEM (error bars).
(0.09 MB TIF)

Figure S2 Mouse ataxin-3 conserves the deubiquitinating activity observed for the human ataxin-3. A) Scheme of the recombinant His-tagged proteins used in this study: mATX3, the full-length protein; mATX3:C14A, the full-length protein carrying the C14A point mutation; mATX3:jos, the Josephin domain of mATX3; mATX3:jos:C14A, the Josephin domain with the C14A mutation; and mATX3:UIMs, the C-terminal of mATX3 containing the three UIMs. B) Polyubiquitin immunoblotting showing the deubiquitinating activity of each used protein. mATX3, as well as its Josephin domain for itself (mATX3:jos) were able to cleave both K48 and K63-linked polyubiquitin chains, preferentially with two or more ubiquitins. The mutation of the catalytic cysteine in these two proteins (mATX3:C14A and mATX3:jos:C14A) abolished their DUB activity. The C-terminal of mATX3 containing the UIMs (mATX3:UIMs) is not capable to hydrolyse polyubiquitin chains.
(0.60 MB TIF)

Table S1 Proteins showing altered levels upon *Mjd* siRNA in undifferentiated C2C12 cells (Day 0). Cut-off (sketched line): proteins with fold changes ≥20% (1.2< ratio <0.8) considering a statistical significant p-value <0.05. Functional networks: (A) cellular movement; (B) drug metabolism; (C) protein synthesis; (D) skeletal and muscular system development and function, tissue morphology; (E) cellular development, embryonic development, cellular assembly and organization; (F) small molecule biochemistry, gene expression.
(0.06 MB PDF)

Table S2 Primers used in this study.
(0.02 MB PDF)
