# Supplementary material for: Ataxin-3 Plays a Role in Mouse Myogenic Differentiation through Regulation of Integrin Subunit Levels
Source: PLoS One. 2010 Jul 23;5(7):e11728. doi: 10.1371/journal.pone.0011728 (PMC2909204; doi:10.1371/journal.pone.0011728)
Supplement: Materials and Methods S1 — (0.02 MB PDF) [file pone.0011728.s001.pdf]

MATERIALS AND METHODS S1

Constructs and mutagenesis

The cDNA fragments encoding the full-length mouse ATX3 protein (mATX3, 1-355aa), the josephin domain (jos, 1-215aa) and the C-terminal (UIMs, 215-355aa) were amplified by PCR, from the pMjd1 plasmid using, respectively, the primer pairs including the 5' and 3' attB site-specific recombination sequences (Gateway Cloning System): attB1Mjd/attB2Mjd, attB1Mjd/attB2Mjd:Jos, and attB1Mjd:UIMs/attB2Mjd (Supplemental Table 2). To obtain the pDONR207Mjd:C14A and pDONR207Mjd:jos:C14A plasmids a point mutation was introduced, corresponding to the alteration of the cysteine for an alanine residue at position 14, into the pDONR207Mjd and the pDONR207Mjd:jos plasmids using the mutagenesis primer Mjd-mutC14A (Supplemental Table 2), the Pfu Turbo enzyme (Stratagene) and the DpnI endonuclease enzyme according to the published conditions (Makarova O et al., 2000). The recombinant His-tag protein expression plasmids (pDEST17Mjd, pDEST17Mjd:jos, pDEST17Mjd:UIMs, pDEST17Mjd:C14A, and pDEST17Mjd:jos:C14A) were obtained by recombination of the corresponding pDONR207 plasmids with the pDEST17 vector using the Gateway Cloning System (Invitrogen). Glutathione-S-transferase (GST)-tag mATX3 (GST::mATX3) expression plasmid (pDEST15Mjd) was obtained by recombination of the pDONR207Mjd with the pDEST15 vector using the Gateway Cloning System (Invitrogen). The pGEX-5X-1 vector (GE Healthcare) was used for the expression of recombinant GST. The pEGFP:Mjd plasmid was generated by subcloning a *BamHI*-digested PCR product into the *BamHI* restriction site of he pEGFP-C1 vector (Clontech). This PCR product was obtained by introducing the *BamHI* restriction sites, in frame at both 5' and 3' of the *Mjd* cDNA coding region, using the pair of primers 5'MjdBamHI/3'MjdBamHI (Supplemental Table 2), the pDONR207Mjd plasmid as template, and the Expand High Fidelity System (Roche) under the

conditions: 5min at 95ºC, followed by 35 cycles of 1min at 95ºC, 1min at 55ºC, 1min at 72ºC, and ending with 5min at 72ºC. All constructs were confirmed by automated sequencing.

Expression and purification of recombinant His-tagged proteins

The recombinant hexahistidine-tagged mATX3, mATX3:C14A, mATX3:jos, mATX3:jos:C14A, and mATX3:UIMs proteins were expressed in *Escherichia coli* BL21SI cells (Invitrogen) and purified using the HiTrap Kit (GE Healthcare) as previously described for the human enzyme (Gales et al., 2005). The fractions of the proteins mATX3, mATX3:C14A, mATX3:jos, and mATX3:jos:C14A, considered appropriate for further purification, were selected by SDS-PAGE analysis, applied to a HiPrep 26/60 Sephacryl S-200 column (GE Healthcare) equilibrated in buffer A (20mM HEPES (pH7.5), 50 mM NaCl, 5% glycerol, 1mM EDTA, 1mM DTT), and protein was eluted with a flow rate of 0,5mL/min, at 4ºC, being monitored at 280nm. After SDS-PAGE and analytical gel filtration analysis (Superdex 200) analysis, the fractions containing only protein monomers were pooled and concentrated in on Millipore Amicon Ultra-15 concentrators (cut-off 10KDa, Millipore) to 1-2mg/mL. Purified monomeric proteins were diluted into a 20μM working solution and maintained in ice for enzymatic assays and then stored at -80ºC.

GST::mATX3 expression and purification

The recombinant GST::mATX3 protein was expressed in *Escherichia coli* BL21SI cells (Invitrogen Life Technologies) as above mentioned. Cells were harvested, ressuspended in PBS, 200μg/mL lysozime, 1% Triton X-100, 1mM phenylmethylsulphonyl fluoride (PMSF), protease inhibitors (Complete, Roche), and stored at -20ºC. After defrosting, the cell suspension was incubated with agitation at 4ºC, during 1h, sonicated, and after centrifugation the supernatant was collected. The soluble recombinant proteins were purified using

glutathione-sepharose™ 4B beads (GE Healthcare) according to manufacturer's instructions, being eluted in three fractions of 10 mM reduced glutathione in 50mM Tris-Cl pH8.0. The eluted fractions considered appropriate were dialyzed using a dialysis membrane with a cut-off of 14KDa (Medicell) placed in PBS, overnight, at 4ºC. Protease inhibitors (Complete, Roche Diagnostics) and PMSF were added to the dialyzed protein that was using the Bradford reagent (Sigma Aldrich).

De-ubiquitination (DUB) assay

K48 and K63- linked multiubiquitin chains (Biomol, International LP) (1μg) were incubated with purified His-tagged mATX3, mATX3:C14A, mATX3:jos, mATX3:jos:C14A, and mATX3:UIMs proteins (2.5μM) in a final volume of 20μL of DUB assay buffer (50mM HEPES (pH7.4), 500mM EDTA, 1mM DTT, 0.1mg/mL BSA, COMPLETE (Roche Diagnostics), during 15 h at 30ºC. Reactions were stopped by addition of Laemmli sample buffer containing 100mM DTT.
