# Supplementary figures and images for: Ataxin-3 Plays a Role in Mouse Myogenic Differentiation through Regulation of Integrin Subunit Levels
Source: PLoS One. 2010 Jul 23;5(7):e11728. doi: 10.1371/journal.pone.0011728 (PMC2909204; doi:10.1371/journal.pone.0011728)

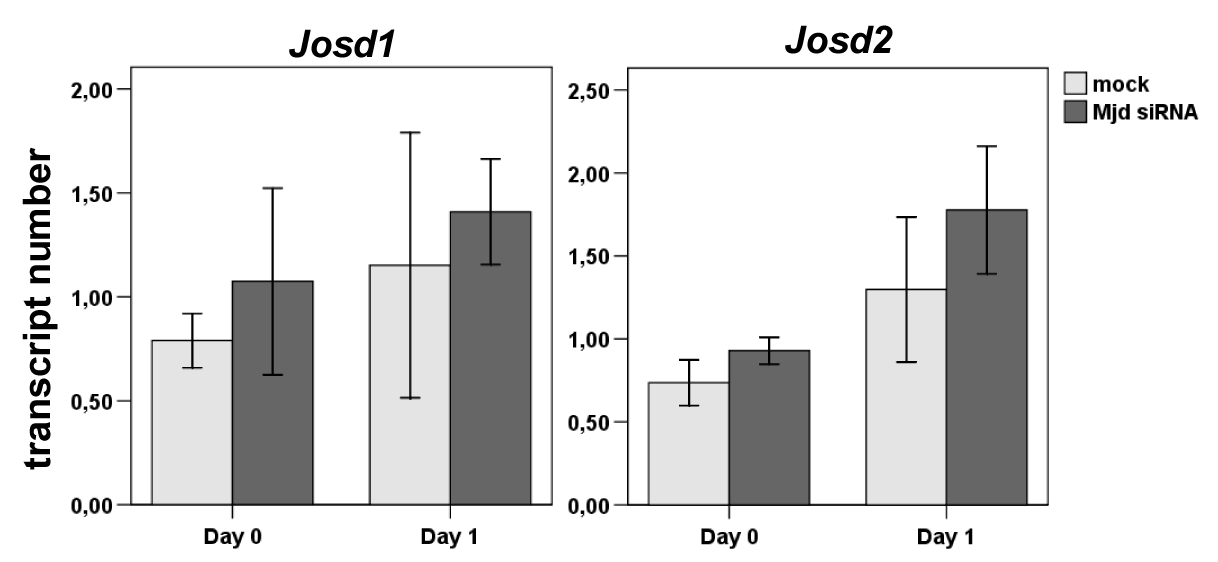

Supplement: Figure S1 — The transcript levels of the genes encoding other josephin-domain containing proteins Josd1, and Josd2 for mock and Mjd siRNA transfected C2C12 cells at Day 0 and Day 1 of differentiation, were measured by quantitative real-time RT-PCR, and showed to be similar. The results were normalised for the Hprt1 gene and correspond to the mean of three independent transfections +/− SEM (error bars). (0.09 MB TIF) [file pone.0011728.s002.tif]

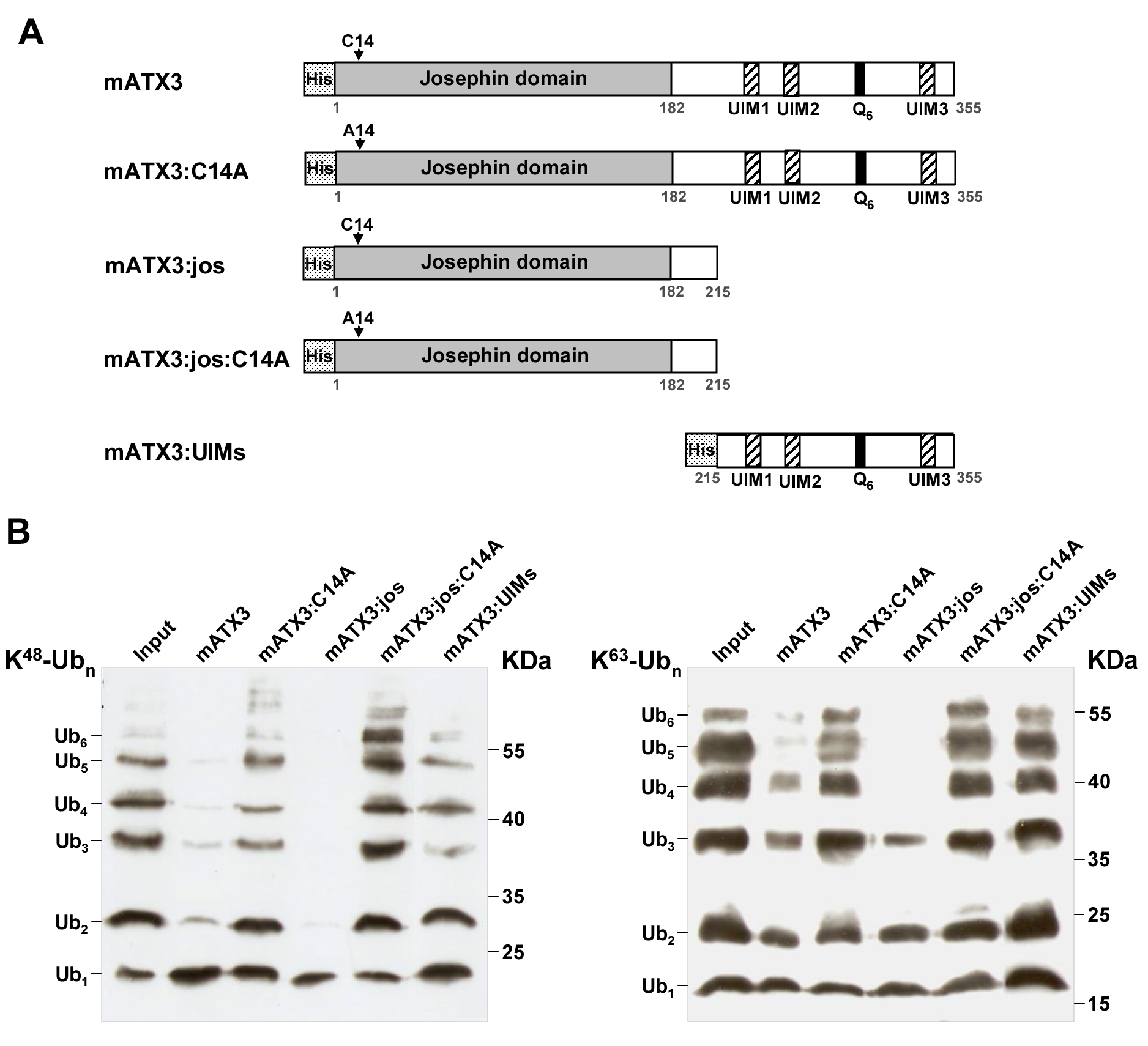

Supplement: Figure S2 — Mouse ataxin-3 conserves the deubiquitinating activity observed for the human ataxin-3. A) Scheme of the recombinant His-tagged proteins used in this study: mATX3, the full-length protein; mATX3:C14A, the full-length protein carrying the C14A point mutation; mATX3:jos, the Josephin domain of mATX3; mATX3:jos:C14A, the Josephin domain with the C14A mutation; and mATX3:UIMs, the C-terminal of mATX3 containing the three UIMs. B) Polyubiquitin immunoblotting showing the deubiquitinating activity of each used protein. mATX3, as well as its Josephin domain for itself (mATX3:jos) were able to cleave both K48 and K63-linked polyubiquitin chains, preferentially with two or more ubiquitins. The mutation of the catalytic cysteine in these two proteins (mATX3:C14A and mATX3:jos:C14A) abolished their DUB activity. The C-terminal of mATX3 containing the UIMs (mATX3:UIMs) is not capable to hydrolyse polyubiquitin chains. (0.60 MB TIF) [file pone.0011728.s003.tif]
