# Supplementary material for: Ataxin-3 Plays a Role in Mouse Myogenic Differentiation through Regulation of Integrin Subunit Levels
Source: PLoS One. 2010 Jul 23;5(7):e11728. doi: 10.1371/journal.pone.0011728 (PMC2909204; doi:10.1371/journal.pone.0011728)
Supplement: Table S1 — Proteins showing altered levels upon Mjd siRNA in undifferentiated C2C12 cells (Day 0). Cut-off (sketched line): proteins with fold changes ≥20% (1.2< ratio <0.8) considering a statistical significant p-value <0.05. Functional networks: (A) cellular movement; (B) drug metabolism; (C) protein synthesis; (D) skeletal and muscular system development and function, tissue morphology; (E) cellular development, embryonic development, cellular assembly and organization; (F) small molecule biochemistry, gene expression. (0.06 MB PDF) [file pone.0011728.s004.pdf]

Table S1. Proteins showing altered levels upon *Mjd* siRNA in undifferentiated C2C12 cells (Day 0). Cut-off (sketched line): proteins with fold changes ≥ 20% (1.2 < ratio < 0.8) considering a statistical significant p-value < 0.05. Functional networks: (A) cellular movement; (B) drug metabolism; (C) protein synthesis; (D) skeletal and muscular system development and function, tissue morphology; (E) cellular development, embryonic development, cellular assembly and organization; (F) small molecule biochemistry, gene expression.

Accession number	Description	Fold change Mjd siRNA/ mock	Functional network
**gi|6680229**	high mobility group box 2	**3.076**	**D**
**gi|31981035**	dynein light chain LC8-type 1	**2.611**	**D**
**gi|31981327**	proteasome (prosome, macropain) subunit, beta type 2	**2.259**	**D**
**gi|84042521**	barrier to autointegration factor 1	**2.233**	**D**
**gi|13542867**	non-metastic cells 1, protein (NM23A)	**1.775**	**A , E**
**gi|114326546**	phosphoglycerate mutase 1	**1.774**	**A**
**gi|37700232**	nucleoside-diphosphate kinase 1	**1.736**	**-**
**gi|84875544**	LIM domain binding 3 isoform c	**1.574**	**B**
**gi|21886811**	S100 calcium binding protein A11 (calizzarin)	**1.559**	**F**
**gi|50263046**	glutathione S-transferase, alpha 2 (Yc2)	**1.540**	**B**
**gi|61098065**	glutathione S-transferase, alpha 1 (Ya)	**1.475**	**-**
**gi|125656173**	acetyl-Coenzyme A carboxylase alpha	**1.470**	**A**
**gi|33859580**	galectin 3	**1.463**	**A**
**gi|115385968**	transportin 1 isoform 1	**1.443**	**B**
**gi|6681079**	cathepsin B preproprotein	**1.440**	**C , F**
**gi|31982030**	Rho GDP dissociation inhibitor (GDI) alpha	**1.401**	**A**
**gi|96975138**	hypoxanthine guanine phosphoribosyl transferase 1	**1.399**	**C**
**gi|56605979**	basic transcription factor 3	**1.391**	**B**
**gi|83921595**	diazepam binding inhibitor isoform 1	**1.387**	**A**
**gi|9790141**	actin related protein 2/3 complex, subunit 3	**1.362**	**A**
**gi|6677833**	S100 calcium binding protein A10	**1.360**	**C**
**gi|61743961**	AHNAK nucleoprotein isoform 1	**1.338**	**E**
**gi|83921612**	thioredoxin domain containing 5	**1.337**	**E**
**gi|13384778**	6-phosphogluconolactonase	**1.336**	**-**
**gi|94378322**	PREDICTED: similar to ribosomal protein L38	**1.331**	**C**
**gi|6680840**	calumenin isoform 1	**1.316**	**F**
**gi|6679439**	peptidylprolyl isomerase A	**1.304**	**B , C**
**gi|10092608**	glutathione S-transferase, pi 1	**1.303**	**B**
**gi|149270774**	PREDICTED: similar to Myoferlin (Fer-1-like protein 3) isoform 2	**1.302**	**D**
**gi|6753364**	cell division cycle 42 homolog	**1.302**	**A**
**gi|6754082**	glutathione S-transferase, alpha 4	**1.297**	**B**
**gi|6997239**	poly(rC) binding protein 2	**1.297**	**-**
**gi|116517336**	four and a half LIM domains 1 isoform 2	**1.287**	**B**
**gi|6681219**	dihydropyrimidinase-like 3	**1.284**	**F**
**gi|19527026**	leucine rich repeat containing 59	**1.272**	**-**
**gi|12963737**	chromosome segregation 1-like	**1.264**	**D**
**gi|21313144**	GTP-binding protein PTD004 isoform a	**1.262**	**-**
**gi|31559990**	torsin family 1, member B	**1.252**	**-**
**gi|6755152**	prolyl endopeptidase	**1.247**	**C**
**gi|22094111**	solute carrier family 2 (facilitated glucose transporter), member 1	**1.246**	**A**
**gi|6681069**	cysteine and glycine-rich protein 1	**1.234**	**E**
**gi|31982186**	malate dehydrogenase 2, NAD (mitochondrial)	**1.225**	**A**
**gi|6754696**	macrophage migration inhibitory factor	**1.223**	**A , F**
**gi|31981302**	annexin A6	**1.221**	**F**
**gi|126012517**	small inducible cytokine subfamily E, member 1	**1.220**	**A**
**gi|6755392**	S100 calcium binding protein A6 (calcyclin)	**1.219**	**C**
**gi|93102409**	fatty acid synthase	**1.219**	**B**
**gi|6754256**	heat shock protein 9	**1.218**	**-**

**gi|113680348**	fascin homolog 1, actin bundling protein	**1.213**	**A**
**gi|6677777**	ribosomal protein L26	**1.206**	**C**
**gi|7106303**	EH-domain containing 1	**1.201**	**E**
**gi|6753320**	chaperonin subunit 3 (gamma)	**-1.251**	**B**
**gi|21281687**	deoxyuridine triphosphatase	**-1.254**	**F**
**gi|126723461**	chaperonin subunit 8 (theta)	**-1.261**	**B**
**gi|94377390**	PREDICTED: similar to insulinoma protein (rig)	**-1.261**	**-**
**gi|31982755**	vimentin	**-1.263**	**A**
**gi|6678419**	tripeptidyl peptidase II	**-1.265**	**C**
**gi|82965849**	PREDICTED: hypothetical protein	**-1.266**	**E**
**gi|116089273**	guanosine diphosphate (GDP) dissociation inhibitor 2	**-1.272**	**D**
**gi|21312298**	serine hydroxymethyltransferase 2 (mitochondrial)	**-1.276**	**C**
**gi|22165392**	non-SMC condensin I complex, subunit D2	**-1.276**	**B**
**gi|13385408**	ribosomal protein L11	**-1.280**	**E**
**gi|31981679**	heat shock protein 1 (chaperonin)	**-1.281**	**A**
**gi|27883844**	small nuclear ribonucleoprotein E	**-1.284**	**D**
**gi|15426055**	coatomer protein complex, subunit beta 1	**-1.289**	**E**
**gi|149269890**	PREDICTED: similar to ribosomal protein L11	**-1.289**	**E**
**gi|41322931**	plectin 1 isoform 6	**-1.300**	**A**
**gi|112363072**	actin related protein 2/3 complex, subunit 2	**-1.303**	**A**
**gi|93102415**	phosphoribosylglycinamide formyltransferase	**-1.310**	**C**
**gi|149234051**	PREDICTED: similar to ribosomal protein L10a	**-1.313**	**-**
**gi|149254252**	PREDICTED: similar to vesicle associated protein	**-1.319**	**B**
**gi|6755364**	RNA polymerase II transcriptional coactivator	**-1.328**	**B**
**gi|33468885**	calcyclin binding protein	**-1.330**	**A**
**gi|33859482**	eukaryotic translation elongation factor 2	**-1.330**	**A**
**gi|47271396**	GNAS complex locus isoform a	**-1.341**	**B**
**gi|6680047**	guanine nucleotide binding protein (G protein), beta polypeptide 2 like 1	**-1.346**	**B**
**gi|27229277**	threonyl-tRNA synthetase	**-1.346**	**-**
**gi|149262992**	PREDICTED: similar to Protein disulfide isomerase associated 6	**-1.390**	**-**
**gi|56699434**	eukaryotic translation initiation factor 4, gamma 1 isoform b	**-1.393**	**A**
**gi|38348472**	adaptor-related protein complex 2, sigma 1 subunit	**-1.406**	**C**
**gi|6677813**	ribosomal protein S8	**-1.424**	**E**
**gi|58037465**	Ribosomal protein L18A	**-1.428**	**E**
**gi|31981534**	proteasome (prosome, macropain) subunit, alpha type 3	**-1.437**	**D**
**gi|6753620**	DEAD/H (Asp-Glu-Ala-Asp/His) box polypeptide 3, X-linked	**-1.447**	**B**
**gi|88853578**	adaptor protein complex AP-1, beta 1 subunit	**-1.470**	**D**
**gi|94395563**	PREDICTED: hypothetical protein isoform 2	**-1.482**	**-**
**gi|6753882**	FK506 binding protein 4	**-1.506**	**B**
**gi|78190507**	exportin 1, CRM1 homolog	**-1.517**	**F**
**gi|25742730**	ribosomal protein L32	**-1.520**	**C**
**gi|10242373**	minichromosome maintenance complex component 7	**-1.527**	**D**
**gi|6755198**	proteasome (prosome, macropain) subunit, alpha type 6	**-1.545**	**D**
**gi|6754616**	mago-nashi homolog, proliferation-associated	**-1.551**	**C**
**gi|31982143**	WW domain binding protein 11	**-1.554**	**-**
**gi|82953132**	PREDICTED: hypothetical protein	**-1.605**	**-**
**gi|8393150**	carboxyl terminal LIM domain protein 1	**-1.607**	**B**
**gi|6755698**	surfeit gene 4	**-1.612**	**E**
**gi|22122515**	AHA1, activator of heat shock 90kDa protein ATPase homolog 1	**-1.621**	**C**
**gi|34328185**	prosaposin	**-1.629**	**D**
**gi|6755206**	proteasome (prosome, macropain) subunit, beta type 7	**-2.225**	**D**
**gi|94378251**	PREDICTED: similar to histone H4	**-2.282**	**-**
**gi|84619697**	sterol O-acyltransferase 1	**-2.555**	**F**
**gi|149269808**	PREDICTED: similar to Eukaryotic translation initiation factor 3, subunit 1 alpha	**-2.557**	**-**
**gi|149272386**	PREDICTED: similar to thymosin beta-4	**-2.630**	**-**
