# Supplementary material for: Ataxin-3 Plays a Role in Mouse Myogenic Differentiation through Regulation of Integrin Subunit Levels
Source: PLoS One. 2010 Jul 23;5(7):e11728. doi: 10.1371/journal.pone.0011728 (PMC2909204; doi:10.1371/journal.pone.0011728)
Supplement: Table S2 — Primers used in this study. (0.02 MB PDF) [file pone.0011728.s005.pdf]

Table S2. Primers used in this study.

Name	Sequence 5'→ 3'	Orientation
**attB1Mjd**	GGGGACAAGTTTGTACAAAAAAGCAGGCTGGATGGAGTCCATCTTCCACGAG	forward
**attB2Mjd**	GGGGACCACTTTGTACAAGAAAGCTGGGTCTTACTTCTTTCGCTCTGCTT	reverse
**attB1Mjd:UIMs**	GGGGACAAGTTTGTACAAAAAAGCAGGCTATATGGCTGATGGGTCGGGCA	forward
**attB2Mjd:Jos**	GGGGACCACTTTGTACAAGAAAGCTGGGTCTTATGCTTCTAAGACGCGCT	reverse
**5'MjdBamHI**	GCCTGGATCCATGGAGTCCATCTTCCACGAG	forward
**3'MjdBamHI**	GCCTGGATCCTTACTTCTTTCGCTCTGCTTT	reverse
**Mjd-mutC14A**	GAAACAAAAAGGCTCACTTGCTGCTCAGCATTGCCTGAA	forward
**Hprt(3)**	GCTGGTGAAAAGGACCTCT	forward
**Hprt(4)**	CACAGGACTAGAACACCTGC	reverse
**Itga5(1)**	GGACGGAGTCAGTGTGCTG	forward
**Itga5(2)**	GAATCCGGGAGCCTTTGCTG	reverse
**Itga7(1)**	GAGCTGGCTGCTGGTGGGCG	forward
**Itga7(2)**	CTCCTTCTGCACGTTAGCTC	reverse
**Josd1(1)**	AAGATGCCAGAGTGGATTGG	forward
**Josd1(2)**	TGATGGGCTTCCACTTCTTC	reverse
**Josd2(1)**	CGGCAACTATGATGTCAACG	forward
**Josd2(2)**	AGCGACACAGGAGAGGGTAG	reverse
